# *GJB2* Gene Mutations in Syndromic Skin Diseases with Sensorineural Hearing Loss.

**DOI:** 10.2174/138920211797904098

**Published:** 2011-11

**Authors:** Sandra Iossa, Elio Marciano, Annamaria Franzé

**Affiliations:** 1 CEINGE-Biotecnologie Avanzate, Naples, Italy; 2 Unità di Audiologia, Dipartimento di Neuroscienze, Università di Napoli “Federico II”, Naples, Italy; 3 Istituto di Genetica e Biofisica “A. Buzzati Traverso”, C.N.R., Naples, Italy

**Keywords:** **GJB2**, connexin 26, skin, hearing loss.

## Abstract

The *GJB2* gene is located on chromosome 13q12 and it encodes the connexin 26, a transmembrane protein involved in cell-cell attachment of almost all tissues. *GJB2* mutations cause autosomal recessive (DFNB1) and sometimes dominant (DFNA3) non-syndromic sensorineural hearing loss. Moreover, it has been demonstrated that connexins are involved in regulation of growth and differentiation of epidermis and, in fact, *GJB2* mutations have also been identified in syndromic disorders with hearing loss associated with various skin disease phenotypes. *GJB2* mutations associated with skin disease are, in general, transmitted with a dominant inheritance pattern. Nonsyndromic deafness is caused prevalently by a loss-of-function, while literature evidences suggest for syndromic deafness a mechanism based on gain-of-function. The spectrum of skin manifestations associated with some mutations seems to have a very high phenotypic variability. Why some mutations can lead to widely varying cutaneous manifestations is poorly understood and in particular, the reason why the skin disease-deafness phenotypes differ from each other thus remains unclear. This review provides an overview of recent findings concerning pathogenesis of syndromic deafness imputable to *GJB2* mutations with an emphasis on relevant clinical genotype-phenotype correlations. After describing connexin 26 fundamental characteristics, the most relevant and recent information about its known mutations involved in the syndromic forms causing hearing loss and skin problems are summarized. The possible effects of the mutations on channel expression and function are discussed.

## 1 INTRODUCTION

Sensorineural hearing loss is a very heterogeneous disorder showing different pattern of inheritance and involving a multitude of different genes. Several forms of hearing loss have been imputated to connexins mutations and prevalently to connexin 26 (Cx26) codified by the *GJB2* gene (gap junction protein, beta 2).

**Fig. (1):**
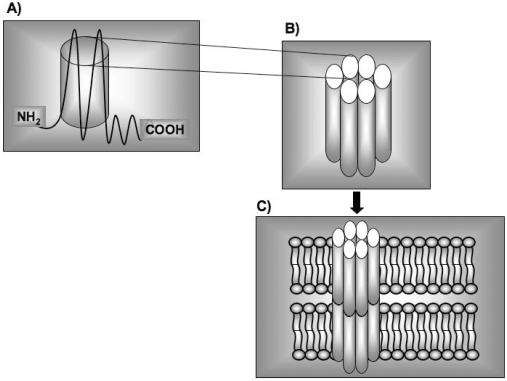
**Schematic structure of connexins, hemichannels (connexons) and gap junctions**. **A**) Connexins are integral membrane proteins involved in many biological functions. **B**) Connexons are constituted by six connexins wich are joined to form in their center an aqueous pore that extends connecting the cytoplasms of the two cells. **C**) The gap junction units are made up of twelve copies of the connexin molecules arranged into two hexamers (connexons), one associated with each cellular membrane and providing a pathway for the transfer of ions and other small molecules between the cells.

Up to now 21 connexins have been established in humans [1], each coding for a transmembrane protein with the same protein topology. Connexins oligomerize with five other connexin molecules to form a connexon (Fig. **1A-B**). Connexins are synthesized in the endoplasmic reticulum (ER) and oligomerize in the ER/Golgi or trans-Golgi network to form connexons [2]. Connexons are subsequently transported to the plasma membrane by vesicular carriers travelling along microtubules. Connexons in adjoining cells fuse through disulfide bonding to form gap junctions (GJ) (Fig. **1C**) [3]. The combination of several connexins lead to diverse connexons and GJ channels with different properties according to the needs of each cell type [4]. Connexins are, in fact, expressed throughout the body and most cells express more than one type of connexin, thus connexons may either stem from a single species (homomeric) or different Cx (heteromeric) and depending on the compatibility of interacting connexons. This diversity is amplified at the level of intercellular channels, which can be formed by similar (homotypic channels) or different homomeric connexons (heterotypic channels), or two heteromeric Cx (heteromeric channels) [5]. Newly formed connexons tend to move laterally towards existing GJ channels, thus forming large gap junction plaques. Depending on the cell type and the connexin expressed, connexons can function as hemichannels, forming a direct transmembrane communication pathway, enabling the permeation of ions, but also of small metabolites such as ATP, cAMP, IP3 and glutamate. Connexons can thus act as secretory pathways and mediate a paracrine signalling. However, they generally must be tightly regulated to avoid loss of metabolites and of ionic gradients across the membrane. In fact, aberrant opening of hemichannels usually leads to cell death, and most connexons remain in closed conformation in the physiological state. GJ channels instead, will enable a direct communication pathway between the cytoplasms of adjacent cells mediating a direct exchange of ions but also of small molecules up to 1 kDa between the cytoplasms of adjacent cells [6].

According to structural similarity, connexins are divided into two major groups: connexins alpha and beta [7, 1]. GJ appeared to be highly selective as many connexins are restricted in their capability to form functional channels with other members of this family especially when it concerns the connexins from alfa and beta groups [7].

Several connexins are involved in human pathologies [6]. It is actually reported that connexins 26, 29, 30, 30.3, 31, 32, and 43 codified respectively by *GJB2*, *GJE1*, *GJB6*, *GJB4*, *GJB3*, *GJB1* and *GJA1* genes are involved in human diseases affecting cochlea and some of them (Cx26, Cx30, Cx30.3, Cx31 and Cx43) have been involved too in syndromic form of hearing loss affecting coclea in combination with epidermis [8-10].

Mutations in the gene *GJB2* (Cx26) are responsible for the major portion of genetic forms of sensorineural hearing loss (SNHL) (http://hereditaryhearingloss.org/). *GJB2* gene (OMIM 121011) mutations account for up to 50% of prelingual recessive non-syndromic deafness, in Caucasian and European populations [11-13] and it is also involved in vestibular dysfunction [14]. Few specific mutations in the *GJB2* gene also underlie autosomal non-syndromic deafness DFNA3 [15]. A digenic model of inheritance has been proposed in some cases; mutations affecting the GJB6 gene, which encodes connexin-30 (OMIM 604418), have been described in association with *GJB2* mutations thereby determining a double heterozygous genotype [16, 17].

## 2 *GJB2* (CX26) STRUCTURE

**Fig. (2):**
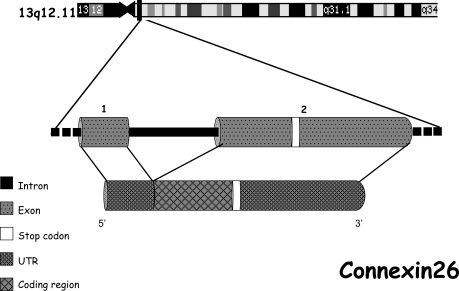
***GJB2* molecular structure and localization.** *GJB2* gene is localized in 13q11 chromosome and it is constituted by two exons: only the exon 2 is a coding one.

**Fig. (3):**
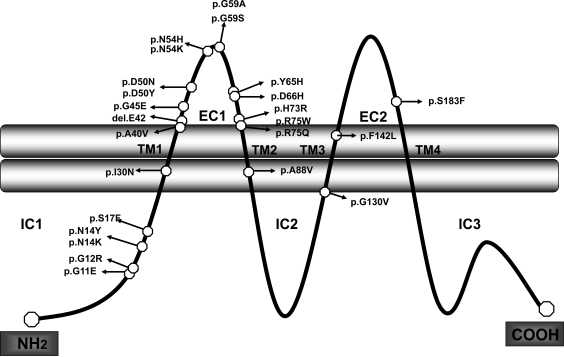
**Connexin26 molecular structure and known syndromic mutations.** Connexins are proteins constituted by four transmembrane domain: TM1-TM4, two extracellular loops: EC1 and EC2, and four intracellular domains: IC1 (NH2-terminal), IC2, and IC3 (COOH-terminal). Known mutations in Cx26 associated with syndromic forms of hearing loss are reported.

*GJB2* gene is located on chromosome 13q12. The structure of the *GJB2* gene, as well the structure of other gap junction genes, is simple (Fig. **2**). An untranslated exon 1 is separated by an intron of 3179 bp length from exon 2, containing the uninterrupted coding region and the 3'-untranslated region (3'-UTR) (Fig. **2**). *GJB2* gene encodes Cx26 protein, that consists of four alfa-helical transmembrane domains (TM1–TM4), two extracellular loops (EC1 and EC2), a cytoplasmic aminoterminal domain (IC1), a cytoplasmic loop (IC2) between TM2 and TM3, and a carboxy-terminal (IC3) domain [18] (Fig. **3**). The two latter domains are characteristic of each connexin, while the membrane spanning and the extra cellular domains are highly conserved among the entire protein family [7, 19]. Under physiological conditions the extra cellular portions of connexons interact with connexons of opposing cells in the intercellular space to complete functional active channels. The cytoplasmic loop and the N-terminus of the protein located at the cytoplasmic side of the channel pore are involved in voltage and ion gating and are thought to be essential for the regulation of channel selectivity [20]. 

## 3 CONNEXIN EXPRESSION IN COCHLEA AND EPIDERMIS

Several connexin are expressed in the cochlea, but the most abundant expression was found for Cx26 and Cx30 proteins which co-localize and can form heteromeric channels. They are expressed in nonsensory epithelial cells among which hair cells are dispersed and connective tissue cells at more distal locations to the hair cells. Despite several studies their function in inner ear is poorly understood. In these cells it has been supposed that gap junctions are important for maintaining the endocochlear potential being involved in recycling endolymphatic K+ ions from the sensory hair cells back to the endolymph [21]. Data suggest that the role of gap junction communication in the cochlea may not be limited to K+ recycling and it has been shown alteration in biochemical coupling, thus proposing that metabolite transfer between cells is the determinant role of gap junction for K+ homeostasis of the inner ear [22, 23].

In the epidermis several connexins are expressed in a partially overlapping pattern. Noteworthy, non-differentiated basal keratinocytes express alfa connexins, while beta connexins prevail in differentiated keratinocytes. Cxs known to be expressed in human epidermis include Cx26, Cx30, Cx30.3, Cx31, Cx31.1, Cx37 and Cx43 [6, 24, 25]. In human skin, only the expression patterns of Cx43 and Cx26 have been studied in detail to date. Cx43 is expressed in interfollicular epidermis, particularly in the spinous and granular cell layers, in sebaceous glands and in hair follicles. Cx26 is present in hair follicles, and in eccrine sweat glands and ducts, but it is much less abundant in normal epidermis, being seen mainly in the skin of palms and soles [26, 27]. 

Cx26 plays an important role in the regulation of both keratinocyte proliferation and differentiation even though it is barely expressed in normal adult epidermis in humans and in rodents [7]. Cx26 expression is induced during wound healing, psoriasis, and skin hyperplasia stimulated by tumor promoters. In hyperplastic proliferating epidermis, Cx26 is co-expressed with Cx43 typical for basal and suprabasal keratinocytes. As Cx26 and Cx43 can not form permeable gap junctions, their co-expression may alter the gap junctional communication between keratinocytes and induce proliferation [28, 29].

## 4 SYNDROMIC FORMS IMPUTABLE TO *GJB2* MUTATIONS

*GJB2* mutations, as well as other connexins, have been reported as causative for several syndromic forms of hearing loss associated to skin problems. The *GJB2* mutations are reported in Table **1**. 

All these syndromic forms have an autosomal dominant pattern of transmission. As well as for the hearing loss, the clinical phenotypes associated have a large variability, the type and the severity of the skin disorders caused by Cx26 mutations are very heterogeneous and sometimes are present only features of incomplete syndrome (Table **2**). The phenotypic variability is observed even among carriers of the same connexin mutation [30-33] (Table **2**).

Below is reported a brief description for each syndrome: 

### A Keratitis-Ichthyosis-Deafness (KID) Syndrome

KID syndrome is a rare congenital ectodermal disorder, characterized by the presence of skin lesions, mild to profound sensorineural hearing loss, and vascularizing keratitis that can result in progressive decline of visual acuity and may eventually lead to blindness. The skin lesions, described as erythrokeratoderma are not restricted to particular regions of the body and show marked ichthyosis with increased susceptibility to mucocutaneous infection sometimes fatal in the neonatal period [34]. Additional phenomena are dystrophic nails, dental abnormalities and scarring alopecia. Sometimes, it is reported increased carcinogenic potential [35-37].

### B Hystrix-Like Icthyosis Deafness Syndrome (HID)

HID deafness syndrome is similar to KID syndrome and displays all of the common features of KID. Symptoms are bilateral hearing loss and spiky hyperkeratotic masses which cover the whole body though the palms and soles are less badly affected. It can be differentiated from KID syndrome which also has symptoms of deafness and ichthyosis by the different distribution of hyperkeratosis. Actually, it is supported the idea that these two syndromes might represent a single form of syndromic deafness with a heterogeneous phenotype. Combined with the similarities between Vohwinkel syndrome (VS), Bart–Pumphrey syndrome (BPS) and palmoplantar keratoderma (PPK), the emerging view is that there are two broad types of skin disorder associated with syndromic deafness: the VS–BPS–PPK group and the KID–HID group [38]. 

### C Palmoplantar Keratoderma with Deafness Syndrome 

Palmoplantar keratoderma is another syndromic complication of deafness (mild to profound SNHL). Hereditary palmoplantar keratodermas (PPK) comprise a clinically and genetically heterogeneous group of genodermatoses, which share impaired epidermal differentiation resulting in prominent palmoplantar hyperkeratosis. Classically, keratodermas have been separated according to their clinical appearance into diffuse, focal, and as a feature of ectodermal dysplasias and many other syndromes [39].

### D Vohwinkel Syndrome

Vohwinkel sindrome is another skin disease associated with SNHL. The skin problem is characterized by disturbed epidermal differentiation manifested by hyperkeratosis especially on the palms and soles (keratoderma), which, in the case of VS, often becomes mutilating with starfish-shaped proximal extensions and hyperkeratotic bands around the fingers, so-called pseudoainhum, sometimes leading to auto-amputation [40,41]. 

### E Bart–Pumphrey Syndrome 

Bart–Pumphrey syndrome, is a rare autosomal dominant disorder characterized by congenital SNHL, palmoplantar hyperkeratosis, knuckle pads, and leukonychia (nail thickening and crumbling) [42-44]. 

## 5 FUNCTIONAL STUDIES

The properties of several specific mutations in connexins involved in ear-skin problems have been investigated in more detail in several studies principally at a cellular level, using either transfected mammalian cells or Xenopus oocyte. These studies give first of all several information about dominant inhibition on wild type connexins [9,45-50]. The more recent and convincing study on dominant inhibition has been realized by Zhang [51] transfecting HeLa cells stably expressing wild type with three Cx26 mutants associated with hearing loss and palmoplantar keratoderma (p.G59A, p.R75Q, and p.R75W). All mutants co-localized and coimmunoprecipitated with wild type Cx26, indicating that they interact physically, moreover, these mutants inhibited the transfer of calcein in cells stably expressing Cx26, demonstrating that they each have dominant negative effects on wild type Cx26. Functional studies gives also several information on connexin trafficking and presence and properties of hemichannels and gap junctions (Tables **3** and **4**). 

## 6 DISCUSSION

It has been demonstrated that connexins have a great importance in many human biological processes. Connexins are expressed in several tissues, but in many cases their effects are restricted to particular organs and mutations in a connexin can affect one organ expressing this protein, but these mutations do not affect other tissues expressing the same protein. The precise mechanism for this selectivity for organ involvement is actually unknown and it can only partially be explained by redundancy in connexin expression or by a specific regulation at the transcriptional level as an alternative splicing [52]. Several data have highlighted, in particular, the importance of some connexins in the cochlea and in the skin. The essential role of connexins in the human cochlea and skin was, in fact, evident from association between mutational changes, deafness [12, 53] and/or skin problems. A chief role in deafness and skin pathologies is carried out by the *GJB2* gene. In fact, mutations in the *GJB2* gene coding for Cx26 can cause a variety of deafness and hereditary hyperproliferative skin disorders in humans. The majority of these mutations, causative of a non–syndromic form of hearing loss, are inherited in an autosomal recessive manner, but several mutations have been also associated with dominantly inherited forms both non-syndromic and syndromic. 

In syndromes associated to *GJB2* mutations (Table **1**) are affected specifically the ear and the epidermis. The lack of associated skin disorders in cases of nonsyndromic SNHL shows that the function and development of the epidermis is not affected by the simple loss of Cx26 function as in the case of homozygous c.35delG patients. In this case a likely explanation is that one or more of the several other connexins expressed in epidermis can compensate for homozygous loss of Cx26. However, while other connexins may compensate for loss of Cx26 from epidermis (but not in the inner ear), they cannot overcome the effects of the dominant mutations. Thus, the Cx26 mutations that can cause syndromic deafness associated with skin disease must show some type of alteration of function, but the mechanisms whereby Cx26 mutation leads to pathological changes remain to be elucidated. 

Phenotype associated to identified mutations is largely variable, broad clinical patterns emerge for the wide range of clinical manifestations and rates of progression (Table **2**). Sometimes the clinical pattern is very serious, even fatal (p.G45E and p.A88V mutations) (Table **2**). A high phenotypic variability has been observed in close aminoacids (p.G11E, p.G12R), sometimes giving also origin to different syndromes (p.A40V: KID and Delta E42: PPK) (Table **2**). It has been observed a variable expressivity also for different mutations in the same amino acid: p.N14K- p.N14Y, p.G59A- p.G59R- p.G59S, p.R75W- p.R75Q. [32, 54]. The amino acid N14 is near two of the residues mutated in Keratitis-like ichthyosis deafness (KID) syndrome (p.G12R and p.S17F), yet the phenotype associated with p.N14K strongly differs from the KID phenotype, having a phenotype more similar to Clouston syndrome (caused by mutations in GJB6). However, a different mutation at the same location, p.N14Y, was reported to cause a disorder similar to KID. p.G59A and p.G59R have been indicated as causative of PPK, while p.G59S has been indicated as causative in an atypical form of BPS as well as in an atypical form of Vohwinkel (Table **2**). One hypothesis to explain the phenotypic differences in these cases may be that distinct substitutions cause different conformational changes to the protein, each with unique consequences for its behaviour. Interestingly, for the p.R75Q mutation, sometimes a  palmoplantar keratoderma phenotype does not consistently occur with the hearing loss giving origin to a non-syndromic form of hearing loss. This high phenotypic variability observed, even among carriers of the same connexin mutation [32, 55] (Table **2**) it has been observed also for p.G45E and p.G130V mutations. The p.G45E mutation, reported as causative of a syndromic fatal form of KID (Table **2**), is however, the third most common *GJB2* mutation (16% of disease alleles) in Japanese patients with autosomal recessive non-syndromic HL [56]. This finding suggests different modes of action of the same *GJB2* mutation depending on the genetic background. Instead, p.G130V is reported as causative of two different syndromic forms: PPK and Vohwinkel (Tables **1** and **2**) [31, 57]. 

To explain the differences observed between the phenotypes it have been assessed several functional studies. Principal results are reported in Table **3** for KID analyzed mutations and in Table **4** for PPK and Vohwinkel analyzed mutations. Several studies in transfected cell systems, such as Xenopus oocytes or HeLa cells, have shown an aberrant hemichannel function for many mutations (Tables **3** and **4**) [58, 59]. This aberrant hemichannel function may contribute to a loss of cell viability and tissue integrity, leading to rapid cell death, probably because of loss of ionic gradient and metabolites. By studying p.G12R and p.D50N mutations it has been determined a cellular death that could be rescued by introduction of Ca^2+^ to the extracellular media during incubation. Moreover, it has been observed [20] by studying the effects of mutations p.G11E and p.D50N that alteration of calcium ion fluxes can result in cell death by necrosis [60]. Free cytoplasmic calcium content is increased in samples with mutated connexins, indicating that a major function of Cx26 is the regulation of calcium flux, whose deregulation leads to predominantly necrotic death. 

Recently, functional consequences of p.N14Y and p.N14K have been assessed, using fluorescently labelled proteins and parachute assay, and compared with that of the classical KID mutation p.D50N [61]. These analyses show that p.N14Y and p.N14K and p.D50N have different consequences for protein localization and gap junction permeability (Table **3**). However, the differences between the phenotypes observed cannot be readily explained from effects on protein trafficking or gap junction permeability.

Besides aberrant hemichannel function, another putative mechanism of disease has also been proposed suggesting that the effect on functions can depends by the specific domain altered. Studies on a mutation associated with Vohwinkel syndrome (p.D66H) [62] and the *GJB2* p.R75W missense mutation [63, 64], associated with sensorineural deafness and PPK, both located in EC1, have both a dominant negative effect on Cx26 gap junction assembly. It is thus suggested that alterations in the EC1 domain could impair proper docking of connexons. Moreover, mutations of some specific residues in the IC1 domains of connexins are consistently found in skin disorders (Table **2**). The Glycine at position 11 or 12 of beta-group connexins seems to be of particular importance. The replacement of this glycine by a polar residue has been associated with skin disease in several connexins (Cx26 p.G12R in relation to KID, Cx31 p.G12R, p.G12D Cx30.3, p.G12D in EKV, and Cx30 p.G11R in Clouston syndrome) [59, 65-68]. Thus, it has been hypothesized that this conserved glycine maintains the flexibility of the NT and enables the gating of the channel by this domain.

By analyzing all identified mutations (Table **2**), it could be observed that KID mutations essentially cluster in the first 50 aminoacid (IC1-TM1-EC1 domains) (Table **2**), with the exception of p.A88V (TM2 domain). Mutations in Cx26 causing PPK–deafness syndrome mostly cluster in the EC1 domain of Cx26 (with the exception of p.G130V located in the IC2 domain), but also mutation involved in other phenotypes BPS, Vohwinkel and Clouston syndromes are located in the same domains; so, further studies are necesary to determine if there is a clear correlation domain-involved and phenotype-associated. 

Autosomal dominant mutations of the Cx26 gene may manifest effects on skin, perhaps due to their dominant-negative effect on wild-type Cx26 and other connexins as determined by several studies [9, 45, 62, 63, 69, 70]. Recent studies have, in fact, shown that mutant forms of Cx26 associated with skin disorders have an impact on the function of Cx43 and Cx30 [28, 45, 71, 72]. These results suggest that the precise phenotypes (deafness versus skin disease) resulting from the different dominant Cx26 mutants may depend upon both the nature of the physical interactions between different mutant and different wild-type connexins and upon their relative levels of expression in particular tissues. It is possible that the cell death associated with dominant connexin mutants results from causes other than direct disruption of tissue gap junction networks. Mutant connexins may exhibit entirely novel interactions with other key cellular proteins, reflected by their mislocation within the cell. This could lead to premature cell death independently of gap junction networks. 

Mutations in connexin-associated protein could also explain the phenotypic variability observed among carriers of the same connexin mutation. It is likely that several syndromes here described are in fact multigenic disorders. Direct and in direct protein partners of connexins are still largely unknown. Further studies in this direction may explain the symptomatic variations of connexins diseases.

## Figures and Tables

**Table 1:** Syndromic Forms Imputable to *GJB2* Mutations

Syndrome	Mutations	OMIM code
Keratitis-ichthyosis- deafness (KID)	G11E, G12R, N14K, N14Y, S17F, I30N, A40V, G45E, D50N, D50Y, A88V	148210
Ichthyosis, hystrix-like-deafness (HID)	D50N, D50Y	602540
Palmoplantar keratoderma- deafness (PPK)	Delta E42, N54H, G59A, G59R, H73R, R75Q, R75W, G130V, S183F	148350
Vohwinkel	G59S, Y65H, D66H, G130V	124500
Burt-Pumphrey	N54K, G59S	149200
Unususal mucocutaneous- deafness	F142L	-

**Table 2:** GJB2 Mutations Involved in SHL: Principal Phenotypic Characteristics and Proteic Domains Affected

Mutations	Syndrome	Phenotypes Ear Skin		Protein domain	References
G11E	KID	Profound SNHL	Hyperkeratosis, ocular problems, alopecia	IC1	[20]
G12R	KID	Mild SNHL	Limited hyperkeratosis. Mild ocular problems	IC1	[8,73]
N14K	KID/EKV	Severe SNHL	EKV-like: hypotrichosis, nail dystrophy, mucositis and skin lesions	IC1	[74]
N14Y	KID	Profound SNHL	Hyperkeratosis palms and soles, impetiginous plaques on neck, axilla, perianal areas and occipital area, ocular problems	IC1	[75]
S17F	KID	SNHL	Visual impairment, in one case lethal carcinoma of the tongue; sometimes trichothiodystrophy-like hair abnormalities	IC1	[8,76,77]
I30N	KID	Profound SNHL	Skin necrosis	TM1	[78]
A40V	KID	Profound SNHL	Mild palmoplantar keratoderma, follicular hyperkeratosis, occlusion triad	TM1/EC1 boundary	[79]
Delta E42	PPK	Profound SNHL	Diffuse PPK	TM1/EC1 boundary	[9]
G45E	KID	Profound SNHL; inner ear abnormality: dysplasia of the cochlear and saccular neuroepithelium.	Severe skin lesion infections and septicaemia:fatal form. In japanese population is not fatal: it is responsible only for a recessive non syndromic form of SNHL	TM1/EC1 boundary	[34,56,80,81]
D50N	KID/HID	Profound SNHL and sometimes conductive HL	Photophobia, keratitis, and erythrokeratoderma; sometimes in association with follicular occlusion triad	EC1	[76,82-84]
D50Y	KID/HID	Profound SNHL	Photophobia, keratitis, and erythrokeratoderma	EC1	[85]
N54H	PPK	Profound SNHL	PPK and knuckle pads	EC1	[86]
N54K	BPS	Profound SNHL	PPK, prominent knuckle pads, and leukonychia	EC1	[42]
G59A	PPK	H.F. SNHL	PPK	EC1	[30]
G59R	PPK	SNHL: Mild at L.F.-severe at H.F.	Striate PPK	EC1	[87]
G59S	Atypical BPS or Atypical Vohwinkel	Severe SNHL Profound SNHL	Knuckle pads, PPK/ ichthyosis, massive mutilating keratoderma, proneness to skin cancer	EC1	[88,89]
Y65H	Vohwinkel	Moderate SNHL	Mutilating PPK	EC1	[90]
D66H	Vohwinkel	Moderate to severe SNHL	Mutilating PPK	EC1	[91]
H73R	PPK	Severe progressive SNHL	Focal PPK similar to Vohwinkel with large intrafamilial variability	EC1	[49]
R75W	PPK	Severe to profound SNHL	Variable: generally PPK and some times knuckle pads	EC1/TM2 boundary	[92,93]
R75Q	PPK	Severe to profound SNHL	PPK (not ever present)	EC1/TM2 boundary	[33,55]
A88V	KID	Severe to profound SNHL	Severe skin lesion infections and septicaemia:fatal form	TM2	[94]
G130V	PPK or Vohwinkel	Severe SNHL Profound HL	Mild PPK PPK and skin constrictions	IC2	[31,57]
F142L	Unususal mucocutaneous- deafness	Severe to profound SNHL	Psoriasiform mucocutaneous involvement, inflammation of mucous membranes	TM3	[95]
S183F	PPK	H. F. SNHL	Focal PPK and some times knuckle pads	EC2	[49]

EKV: erythrokeratodermia variabilis; H. F. : high frequency; L. F. : low frequency.

**Table 3:** Altered Properties Identified by Functional Assays for Cx26 KID Mutations

Mutation	Trafficking	Hemichannels	Functional GJ channels	Observations	References
G11E	Altered	Aberrant opening	Absent	Increase of calcium uptake, increase of cellular death by necrosis	[20]
G12R	OK	Increased function	Absent	Increased cell death	[59]
N14K	OK	Increased function	Present, but with loss of voltage gating	Increased cell death	[59]
N14Y	-	-	Absent	Reduced GJ intercellular communication	[75]
S17F	OK	Complete loss of functions	Absent	No increased cellular lethality	[59]
A40V	OK	Leaky hemichannels	Present	Increased cell death	[79,96,97]
G45E	OK	Excessive entry of Ca^2+^	Present	Increased cell death	[79,96,97]
D50N	-	Increased function	-	Increase of calcium uptake, increase of cellular death by necrosis and apoptosis	[20,59]

**Table 4:** Altered Properties Identified by Functional Assays for Cx26 PPK and Vohwinkel Mutations

Mutation	Trafficking	Hemichannels	Functional GJ channels	Observations	References
Delta E42	OK	-	Absent	-	[9,98]
G59A	OK	Heteromeric: inhibition of calcein transfer	Present	-	[52,99]
Y65H	Altered	-	Absent	-	[90]
D66H	Altered	-	Absent	-	[45,49,100]
H73R	Altered	-	-	-	[49]
R75W	OK	Heteromeric: inhibition of calcein transfer	Present	-	[51,99]
R75Q	OK	Heteromeric: inhibition of calcein transfer	Present	-	[51,99]
